# Proton pump inhibitor usage associates with higher risk of first episodes of pneumonia and peritonitis in peritoneal dialysis patients

**DOI:** 10.1080/0886022X.2022.2129064

**Published:** 2022-10-04

**Authors:** Yujing Zhang, Jiao Li, Zijun Chen, Lingling Liu, Xiaojiang Zhan, Fenfen Peng, Qian Zhou, Xianfeng Wu, Yingsi Zeng, Liya Zhu, Yuxin Xie, Xiaochun Lai, Zebin Wang, Yueqiang Wen, Xiaoran Feng, Jianbo Liang

**Affiliations:** a Department of Nephrology, The Second Affiliated Hospital Guangzhou Medical University, Guangzhou, China; b Department of Cardiology, The Second Affiliated Hospital Guangzhou Medical University, Guangzhou, China; c Department of Nephrology, Affiliated Dongguan People’s Hospital Southern Medical University, Guangdong, China; d Department of General Medicine, The Third Affiliated Hospital Sun Yat-sen University, Guangzhou, China; e Department of Nephrology, The First Affiliated Hospital of Nanchang University, Nanchang, China; f Department of Nephrology, Zhujiang Hospital Southern Medical University, Guangzhou, China; g Department of Medical Statistics, Clinical Trials Unit, The First Affiliated Hospital, Sun Yat-sen University, Guangzhou, China; h Department of Nephrology, Affiliated Sixth People’s Hospital Shanghai Jiao Tong University, Shanghai, China; i Department of Nephrology, Jiujiang NO.1 people’s Hospital, Jiujiang, China

**Keywords:** Proton pump inhibitors, peritoneal dialysis, pneumonia, peritonitis

## Abstract

**Background:**

A large number of studies have shown that proton pump inhibitors (PPIs) are associated with infection events. Therefore, we retrospectively evaluated the association of PPI therapy with the occurrence of first pneumonia and peritoneal dialysis(PD)-related peritonitis events in the maintenance PD patients.

**Methods:**

We collected PD patients in two large hospitals from January 1, 2012 to December 31, 2016, and divided them into the PPI group and the non-PPI group. Multivariate Cox proportional hazards models were applied to evaluate the cumulative incidence and hazard ratios (HRs). Inverse probability of treatment weight (IPTW) method was used to adjust for covariate imbalance between the two groups and further confirm our findings.

**Results:**

Finally, 656 PD patients were included for data analysis, and the results showed that PPI usage was associated with an increased risk of pneumonia [HR 1.71; 95% CI 1.06-2.76; *p* = 0.027] and peritonitis [HR 1.73; 95% CI 1.24-2.40; *p* = 0.001]. IPTW-adjusted HRs for the association of PPIs with pneumonia and peritonitis were 1.58 (95% CI:1.18-2.12; *p* = 0.002) and 2.33 (95% CI:1.91-2.85; *p* < 0.001), respectively. Moreover, the competitive risk model proved that under the conditions of competition for other events(including transfer to hemodialysis therapy, kidney transplant, transfer from our research center, loss to follow-up, and death), the differences in endpoints events between the two groups were still statistically significant (*p* = 0.009, *p* < 0.001, respectively).

**Conclusions:**

PPIs was associated with an increased risk of first pneumonia and PD-related peritonitis events in PD patients, which reminds clinicians to be cautious when prescribing acid-suppressing drugs for PD patients.

## Introduction

Infection is one of the most common complications in dialysis patients. According to the National Kidney Registry of the United States, infection has become the second cause of hospitalization and death in patients with kidney disease, and one-quarter of them were due to lung infections [1]. Peritonitis is another common and serious infectious event in peritoneal dialysis (PD) patients, which causes a mortality rate of about 16% [2–7]. In addition, frequent or severe peritonitis can also lead to failure of PD techniques, peritoneal damage or failure, transfer to hemodialysis (HD), and even death [8]. Therefore, it is vital to reduce and prevent infection for PD patients.

Since the advent of acid inhibitor drugs, they have gradually become one of the most widely used drugs in the world. Due to the extremely high frequency of acid inhibitors in clinical application, the adverse effects brought by them have attracted people’s attention. An early study evaluated the correlation between any type of acid inhibitors therapy and the development of hospital-acquired pneumonia (HAP), and found that patients receiving acid suppression therapy were more likely to develop HAP than patients not taking acid inhibitor drugs [9]. Further analysis of the different types of acid inhibitor drugs found that this correlation existed in patients treated with proton pump inhibitors (PPIs), but not in patients treated with histamine-2 receptor antagonists (H2RAs) [10]. In addition, a meta-analysis study has demonstrated that the use of acid inhibitors was a risk factor for enteroperitonitis [11].

A recent questionnaire on outpatient prescriptions for hemodialysis patients in Japan found that because the risk of gastrointestinal bleeding in dialysis patients was higher than that of the general population, clinicians often prescribe acid inhibitor drugs to dialysis patients, of which proton pump inhibitors was one of the most commonly used [12]. As a special population, there are few studies on whether the use of PPI is related to the infection event in PD population. Therefore, we conducted this study to explore the relationship between PPIs and the first infectious events in PD patients.

## Materials and methods

### Study design and participants

This study recruited patients who received PD treatment at two centers from January 1, 2012 to December 31, 2016. Of the 707 patients, 51 were excluded due to the following reasons: younger than 18 years (*n* = 11), maintaining PD for less than 3 months (*n* = 8), and excessive data loss (*n* = 32). Ultimately, this study included 656 patients. The institutional review boards of the two PD centers approved this retrospective study (IRB approval number 2022-hg-ks-01) and exempted informed consent because all our medical records were collected retrospectively.

### Data collection

The demographic data such as center, gender, age, weight, height, comorbidities, and medication history were collected at the start of PD treatment, whereas laboratory parameters were collected within 90 days of PD treatment initiation. We retrospectively collect comorbidities from the medical records, such as hypertension, diabetes, and cardiovascular disease (CVD). The definition of CVD included ischemic heart disease, congestive heart failure, angioplasty, coronary artery bypass surgery, cerebrovascular disease, or peripheral vascular disease. Pneumonia was diagnosed if one of the pneumonia ICD codes was existed and there was evidence of infection on chest radiography or chest CT. The organisms isolated from peritoneal dialysis effluent were tested, and the diagnosis of PD-related peritonitis was based on at least the following two criteria [13]: (1) abdominal pain or cloudiness of PD effluent; (2) white blood cell count in PD effluent >100/μL, with >50% polymorphonuclear leukocytes; or (3) a positive culture from PD effluent.

### Clinic outcomes

During the follow-up period, the main exposure was the use of PPI, and the primary endpoint was the first occurrence of pneumonia and peritonitis. Similar to previous studies, patients who used PPIs continuously for more than 1 week were classified as PPI group, and the remaining patients were classified as Non-PPI group [14, 15]. It is worth noting that only patients who took PPIs before the onset of pneumonia or peritonitis were included in the PPI group. All patients were followed until pneumonia/peritonitis, death, transfer to hemodialysis therapy, kidney transplantation, transfer from two centers, or censoring on December 31, 2017.

### Statistical analysis

Continuous variables were all described as median (25th to 75th percentile), and the differences between the two groups were tested by Mann-Whitney because of their skewed distribution. Categorical data was given as percentages, and the χ^2^ test was used for comparison between groups. In our study, survival was calculated using Kaplan–Meier method and differences between distributions of survival were assessed by log-rank test. An IPTW model was established to evaluate the relationship between the use of PPI and first pneumonia and peritonitis events using the estimated propensity score as weights. Multivariable Cox regression model was constructed to estimate the relationships between the use of PPI and first pneumonia or peritonitis event adjusting for covariates that were associated with events of pneumonia or peritonitis (*p* < 0.05) and potential confounders as judged by our team. Moreover, competitive risk models were performed to explore whether other follow-up endpoint events had an effect on the first event of pneumonia or peritonitis. Forest plots were used to show the differences between PPI treatment and these two infectious events in different subgroups. The SPSS (version 22.0), and R software (version R-3.5.2) were performed for Statistical analyses. *p* < 0.05 was considered statistically significant.

## Results

**Figure 1.**
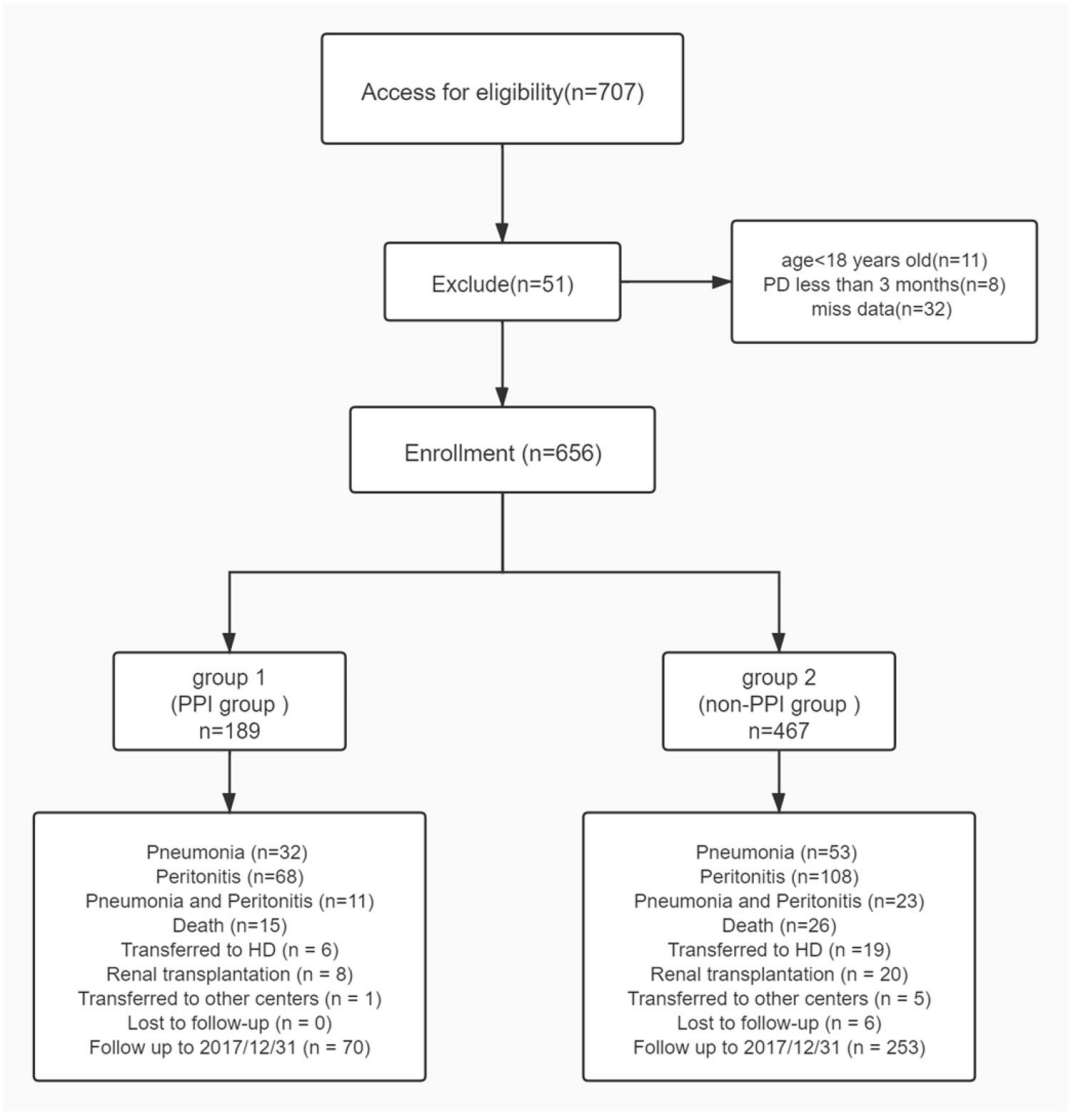
Flow chart- including patient enrollment and outcomes.

Baseline demographic and clinical characteristics of the cohort were given in Table 1, one hundred and eighty-nine patients were exposed to PPIs and 467 patients did not receive PPIs (Figure 1).

**Table 1.** Baseline data characteristics.

	Total (*n* = 656)	Group1 Non-PPI (*n* = 467)	Group2 PPI (*n* = 189)	*p* Value
**No.of C1/C2**	365/469	225/242	39/150	<0.001
**No.of men/women**	367/289	274/193	93/96	0.027
**Demographics**				
Age(y)	53 (43, 63)	52 (42, 62)	57 (44, 66)	0.010
BMI(kg/m^2^)	22.8 (20.7, 25.0)	22.9 (20.7,25.2)	22.6 (20.7, 24.6)	0.385
**Comorbid**				
Hypertension	367 (55.9%)	231 (49.5%)	136 (72.0%)	<0.001
Systolic BP(mmHg)	145 (136, 165)	144 (137, 160)	150 (132, 172)	0.379
Diastolic BP(mmHg)	85 (79, 94)	85 (80, 93)	84 (75, 96)	0.184
Diabetes	164 (25.0%)	106 (22.7%)	58 (30.7%)	0.032
Cardiovascular disease	145 (22.1%)	89 (19.1%)	56 (29.6%)	0.003
Gastrointestinal bleeding	48 (7.3%)	25 (5.4%)	23 (12.2%)	0.002
Smoke	60 (9.1%)	43 (9.2%)	17 (9.0%)	0.932
**Laboratory variables**				
WBC (4.0-10.0, ×10^9^/L)	6.7 (5.6-8.1)	6.7 (5.6, 7.8)	7.1 (5.6, 8.6)	0.054
Hemoglobin (130-175, g/L)	95 (83, 108)	96 (84, 110)	91 (83, 104)	0.047
Albumin (40-55, g/L)	33.0 (29.8, 35.9)	33.3 (30.0, 36.2)	32.5 (28.6, 35.0)	0.006
Creatinine (53-115, umol/L)	780 (559, 999)	769 (550, 963)	816 (599, 1124)	0.028
BUN (3.1-8.8, mmol/L)	19.0 (14.5, 24.6)	19.0 (14.8, 24.7)	19.1(13.6, 23.9)	0.311
Uric acid(150-350, umol/L)	427 (369, 491)	432 (369, 494)	413 (367, 491)	0.232
FBG (3.9-6.1, mmol/L)	4.5 (4.0, 5.6)	4.6 (4.1, 5.6)	4.5 (3.9, 5.6)	0.161
Cholesterol (3.0-5.2, mmol/L)	4.4 (3.8, 5.1)	4.4 (3.8, 5.1)	4.4 (3.8, 5.2)	0.973
Triglycerides (0.5-1.7, mmol/L)	1.4 (1.0, 2.0)	1.4 (1.0, 1.9)	1.5 (1.0, 2.3)	0.133
Sodium (137-147, mmol/L)	141.0 (138.5, 143.0)	141.0 (138.5, 143.0)	141.2 (138.6, 143.1)	0.577
Chlorine (99-110, mmol/L)	100.3 (97.3, 103.0)	100.4 (97.6, 103.2)	99.5 (96.5, 102.9)	0.094
Calcium (2.1-2.5, mmol/L)	2.1 (1.9, 2.2)	2.1 (2.0, 2.2)	2.0 (1.9, 2.2)	0.001
Potassium (3.5-5.3, mmol/L)	3.9 (3.5, 4.5)	3.9 (3.5, 4.5)	3.9 (3.4, 4.4)	0.916
Phosphorus (0.8-1.5, mmol/L)	1.6 (1.2, 1.9)	1.5 (1.2, 1.9)	1.6 (1.2, 2.1)	0.185
Total KT/V	2.3 (1.9, 2.7)	2.3 (2.0, 2.7)	2.2 (1.8, 2.6)	0.186
RRF (mL/min)	9.9 (3.1, 32.4)	7.5 (2.9, 29.7)	17.9 (4.1, 38.5)	<0.001
**Treatments**				
CCB (yes VS no)	547 (83.4%)	386 (82.7%)	161 (85.2%)	0.430
ACEI/ARB (yes VS no)	346 (52.7%)	264 (56.5%)	82 (43.4%)	0.002
EPO (yes VS no)	348 (53.0%)	232 (49.7%)	116 (61.4%)	0.007
Aspirin (yes VS no)	65 (9.9%)	48 (10.3%)	17 (9.0%)	0.618
Insulin (yes VS no)	106 (16.2%)	69 (14.8%)	37 (19.6%)	0.130
Statin (yes VS no )	135 (20.6%)	84 (18.0%)	51 (27.0%)	0.010

All continuous variables are skewed distribution, the values for continuous variables are given as median(P25, P75).

C1: center 1; C2: center 2; BMI: Body mass index; WBC: white blood cell; BUN: Blood urea nitrogen; FBG: fasting blood-glucose; KT/V: K-dialyzer clearance of urea, T-dialysis time, V-volume of distribution of urea; RRF: residual renal function; CCB: calcium channel blocker; ACEI: angiotensin converting enzyme inhibitors; ARB: angiotensin receptor blocker; EPO: erythropoietin.

As shown in Table 1, compared with the non-PPI group, patients in the PPI group were usually older, with higher levels of white blood cells and creatinine, and lower levels of hemoglobin and albumin. The use of ACEI/ARB, EPO, and statins was more common in the PPI group. Among patients receiving PPIs therapy, they were more likely to have a history of hypertension, diabetes, CVD, and gastrointestinal bleeding. During the follow-up period, 85 cases of pneumonia (12.4%) and 176 cases of peritonitis (26.8%) occurred. Among them, the first pneumonia event was 32 cases (16.9%) in the PPI group and 53 cases (11.3%) in the non-PPI group; while 68 (36.0%) and 108 (23.1%) developed first peritonitis in PPI and non-PPI groups, respectively.

**Figure 2.**
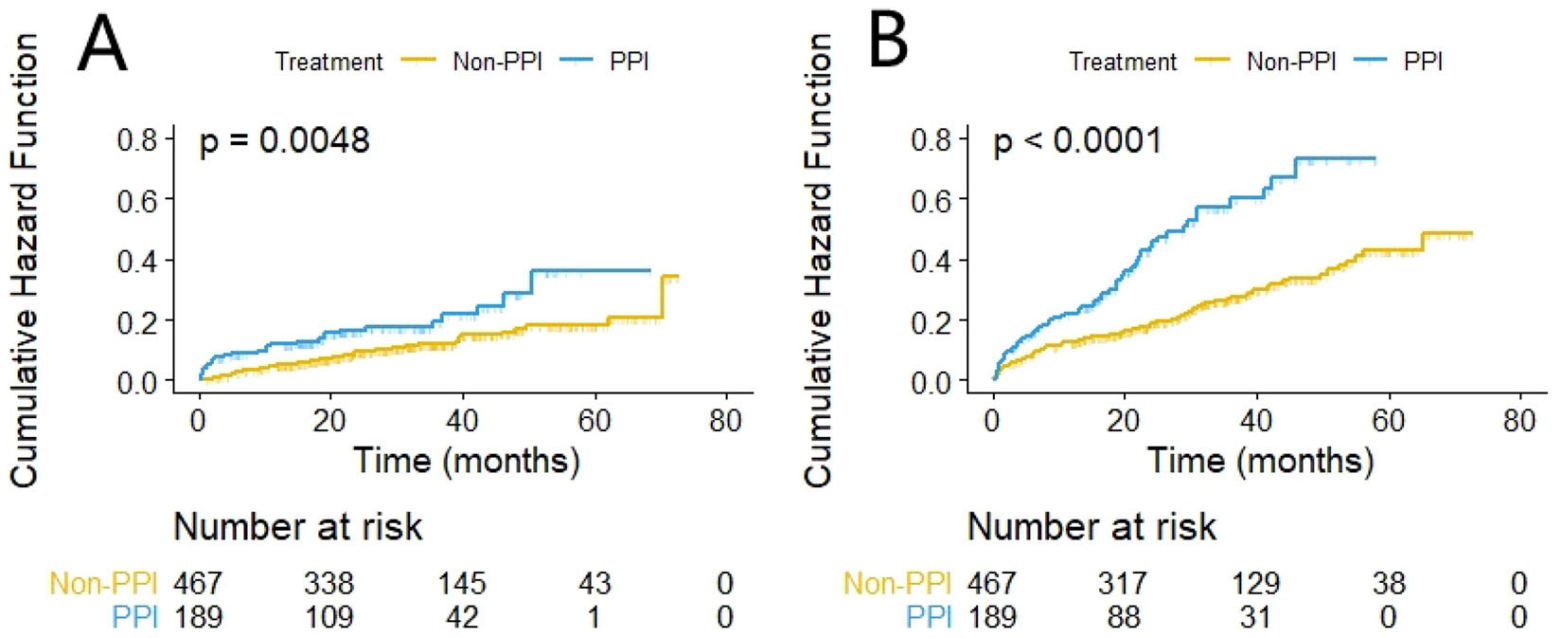
Comparison of the first occurrence of the Pneumonia disease (A) and the Peritonitis disease (B), using Kaplan–Meier method, between patients in the PPI group (*n* = 189) and the non-PPI group (*n* = 467).

The cumulative incidence of first pneumonia and peritonitis events was represented by the Kaplan–Meier curve and tested by log-rank test. The results showed that compared to the non-PPI group, the cumulative incidence of first pneumonia and peritonitis in the PPI group was higher (log-rank test: *p* = 0.0048, *p* < 0.001, respectively)(Figure 2).

The Cox proportional hazards model was used to test the difference in the results of the first infectious event between the two groups. In the unadjusted cohort, after including possible confounding factors related to pneumonia or peritonitis, the use of PPI was associated with the occurrence of the first pneumonia event (HR 1.71; 95% CI 1.06-2.76; *p* = 0.027) and the first peritonitis event (HR 1.73; 95% CI 1.24-2.40; *p* = 0.001) (Table 2).

**Table 2.** The relationship between PPI and the Pneumonia disease and Peritonitis disease.

	Pneumonia		Peritonitis	
	HR(95%CI)	*p* Value	HR(95%CI)	*p* Value
**Unadjusted**	1.87 (1.20-2.92)	0.006	2.07 (1.53-2.82)	<0.001
**Model1**	2.33 (1.44-3.75)	0.001	1.91 (1.38-2.65)	<0.001
**Model2**	1.66 (1.04-2.66)	0.035	1.66 (1.20-2.29)	0.002
**Model3**	1.71 (1.06-2.76)	0.027	1.73 (1.24-2.40)	0.001
**IPTW**	1.58 (1.18-2.12)	0.002	2.33 (1.91-2.85)	<0.001

**Note:** Reference group is Non-PPI group.

**Model 1:** center, sex, age, BMI.

**Model 2:** Model 1 plus Comorbid conditions ((HBP, DM, Cardiovascular diseases, Gastrointestinal bleeding), Medications (CCB, statin, EPO, Insulin, Aspirin).

**Model 3:** Model 2 plus albumin, BUN, p, KTV, RRF.

PPI: proton pump inhibitor; HR: hazard ratio; CI: confidence interval; IPTW: inverse probability of treatment weighting; HBP: high blood pressure; DM: diabetes mellitus; CVE: cardiovascular events; EPO: erythropoietin; CCB: calcium channel blocker; BUN: blood urea nitrogen; KT/V, K-dialyzer clearance of urea, T-dialysis time, V-volume of distribution of urea; RRF: residual renal function.

**Figure 3.**
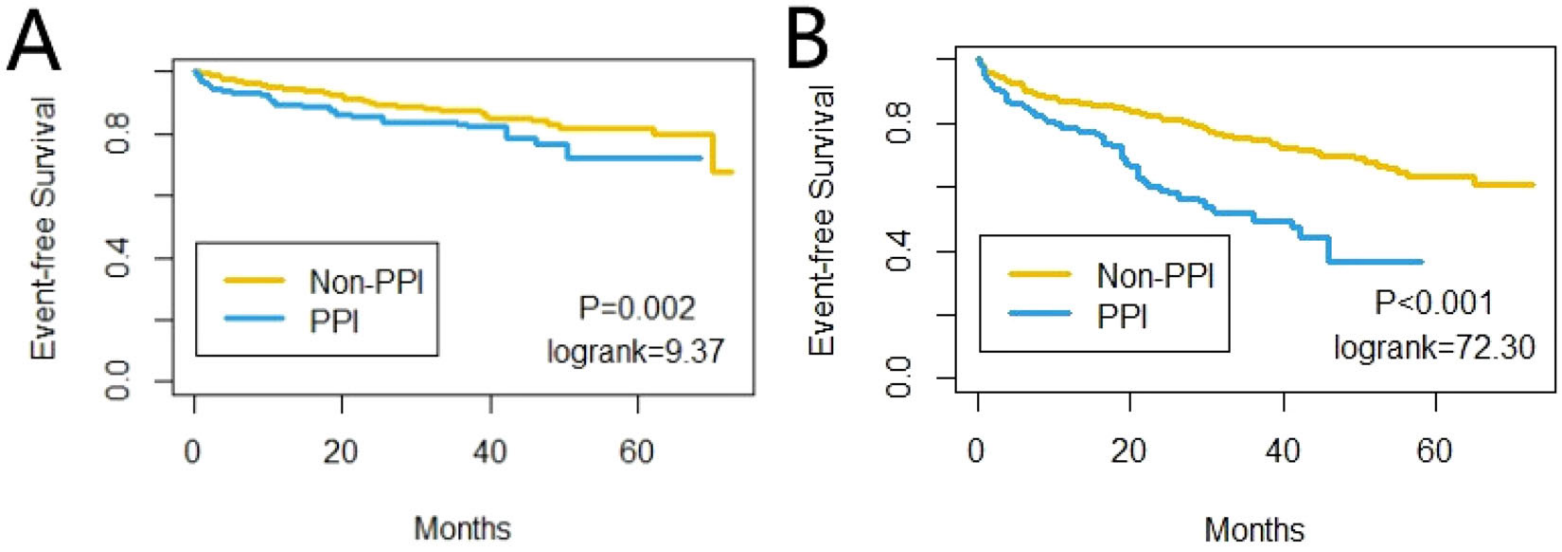
Comparison of the first occurrence of the Pneumonia disease (A) and the Peritonitis disease (B), adjusted using inverse-probability of treatment weighting, between patients in the PPI group and the non-PPI group.

Similar to the results of the unadjusted model, the IPTW-adjusted model also showed that PPIs was related to the occurrence of the first pneumonia events (HR 1.58, 95% CI [1.18-2.12], *p* = 0.002) and the first occurrence of peritonitis (HR 2.33, 95% CI [1.91-2.85], *p* < 0.001) (Table 2). In addition, the cumulative incidence of first pneumonia and peritonitis events after IPTW-adjusted was also statistically different between the two groups (log-rank test: *p* = 0.002, *p* < 0.001, respectively) (Figure 3).

**Figure 4.**
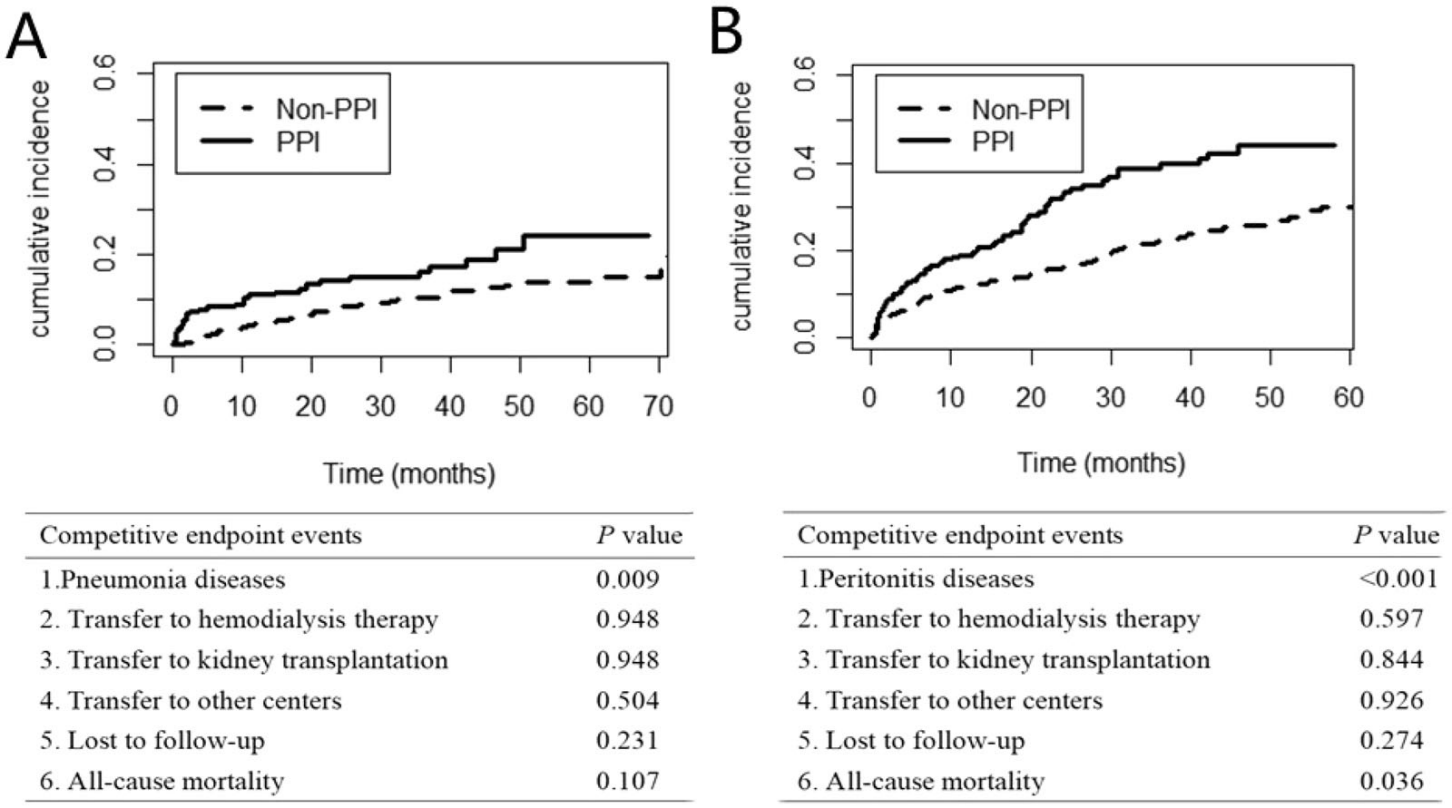
(A) Estimated cumulative incidence curves between the Pneumonia diseases and other competing events.(B) Estimated cumulative incidence curves between the Peritonitis diseases and other competing events.

In the competitive risk model, the difference in the cumulative incidence function (CIF) of first pneumonia and peritonitis event between the PPI group and non-PPI group was statistically significant (*p* = 0.009, *p* < 0.001, respectively), and the difference in other endpoints was not statistically significant (Figure 4).

**Figure 5.**
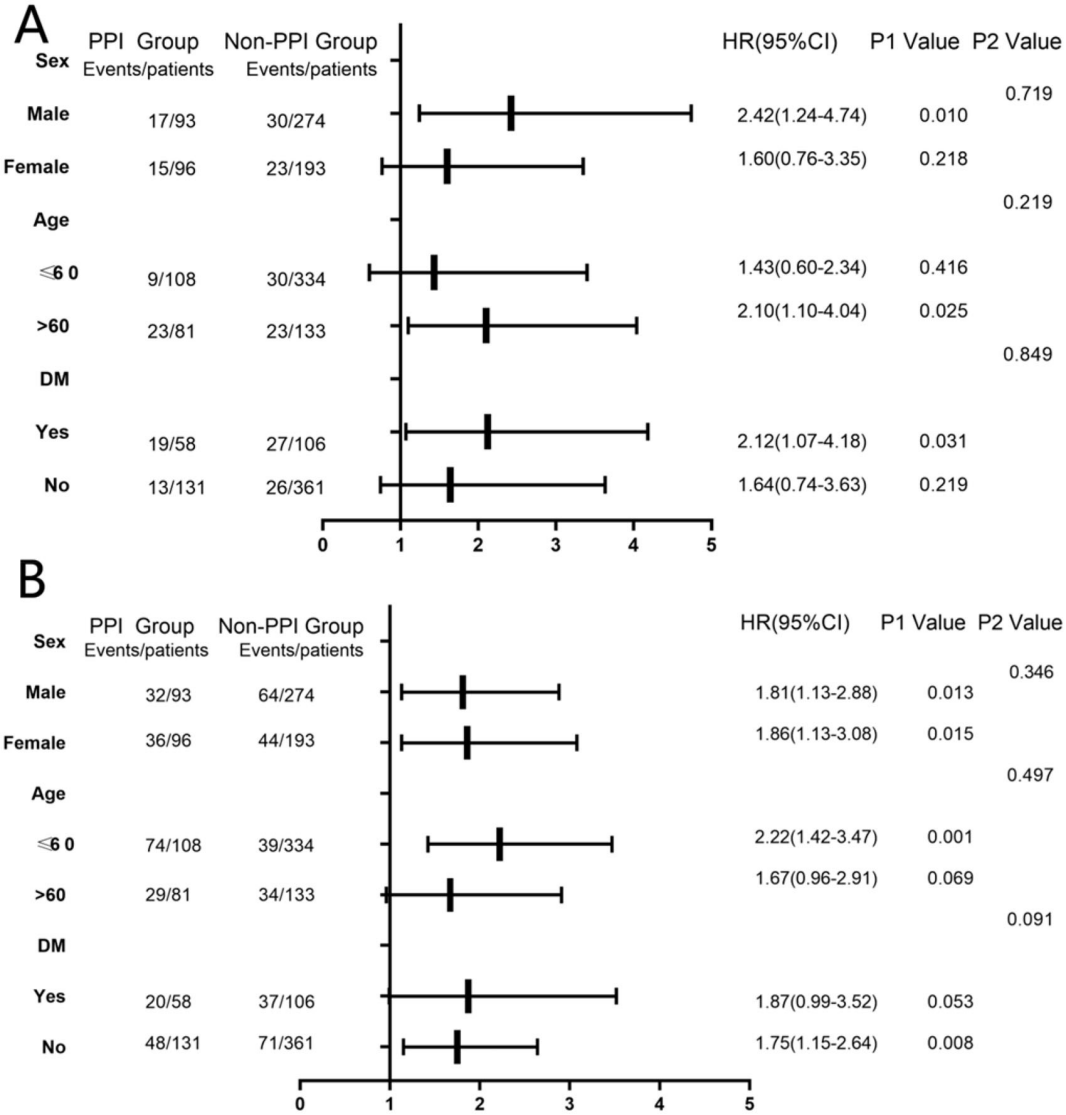
(A) Forest plot of relationship between PPI and the pneumonia diseases in different subgroups. (B) Forest plot of relationship between PPI and the peritonitis diseases in different subgroups. The P1 value corresponds to the relationship between PPI and the pneumonia diseases(A) or peritonitis diseases(B) in different subgroups. The P2 value corresponds to the interaction test between the PPI and the subgroups variable of interest. **Adjusted model:** center, sex, age, BMI, the history of hypertension, diabetes mellitus, cardiovascular events and gastrointestinal bleeding, the use of statin, Insulin, Aspirin, EPO and CCB, albumin, BUN, p, KTV, RRF (In particular, the adjustment model should exclude its own factors in different subgroups. For example, in the age subgroup, the adjustment model does not include age), HR: hazard ratio; CI: confidence interval.

We investigated the relationship between PPIs and the first occurrence of pneumonia and peritonitis in different subgroups of interest, including gender, age, and history of diabetes. Used COX analysis to explore whether there were statistical differences in the subgroups, and expressed them by forest plot. After analysis, no interaction was found in these subgroups (Figure 5).

## Discussion

In the PD population, PPIs was mainly used to treat or prevent peptic ulcers, gastrointestinal bleeding, severe gastrointestinal reactions during dialysis, and preventive stomach protection with anti-platelet drugs or non-steroidal drugs [16, 17]. In this retrospective observational study, we used the IPTW method to test the effect of PPI usage on the first episode of pneumonia and peritonitis in PD patients. Our results showed that in PD patients, PPIs was associated with an increased risk of first pneumonia and peritonitis events.

Existing studies on PPIs and pneumonia were mainly for the general population, stroke population or people with diabetes, while the research on PPIs and peritonitis was mainly focused on people with hepatic cirrhosis. Few researches have been conducted on this specific population of dialysis. In a large retrospective cohort study of the stroke population in Taiwan, it was found that the use of acid-suppressing drugs was an independent risk factor of pneumonia, and only the use of PPI could increase the risk of chronic stroke-associated pneumonia (SAP) [18]. Lin *et al.* found that PPI usage increased the incidence of pneumonia in patients with type 2 diabetes, and this effect was more significant in patients taking high-dose PPIs [19]. Consistent with previous research results, our findings suggested that in the adjusted model, the risk of first pneumonia events was 1.58 times higher in the PPI group than in the non-PPI group.

The mechanism of PPIs associated with pneumonia may be explained by the following reasons. As we all know, gastric acid acted as an important barrier to prevent pathogens from invading due to its lowering of the PH value of the gastrointestinal tract. PPIs inhibited the secretion of gastric acid mainly by irreversibly binding the H-K-ATPase on the cell membrane of the gastric parietal to inactivate it, thus increasing the pH value of the stomach, which in turn led to the excessive growth and colonization of pathogens [20–22]. And PPIs also reduced the acidity of the upper gastrointestinal tract, which changed the oral flora and further led to respiratory infections [23, 24]. In addition, a basic study in mice has found that the intestinal microbiota acts as a protective mediator during pneumococcal pneumonia, and the gut microbiota enhances the function of primary alveolar macrophages [25]. In a large cohort study of healthy people, it was found that PPI use significantly increased the growth of streptococci [26], while studies on streptococcal pneumonia confirmed that PPIs was associated with an increased risk of CAP caused by streptococcus pneumonia infection [27]. There was also evidence that acid inhibitors may damage immune cell function, including T lymphocytes, neutrophils, or natural killer cells [28–32], which may increase the body’s susceptibility to infection [33].

In patients with liver cirrhosis, the use of PPI was closely related to the occurrence of peritonitis, which has been confirmed in many studies [34, 35]. However, there was less literature on the relationship between acid inhibitors and peritonitis associated with peritonitis in PD population, and the results were conflicting. A single-center retrospective analysis compared the use of acid inhibitors in PD patients with peritonitis and non-peritonitis, and the results showed that only H2RAs, rather than PPIs, was associated with an increased risk of PD-associated peritonitis [36]. Caravaca *et al.* found that the use of gastric acid suppression was an independent risk factor for intestinal peritonitis [37]. A recent Japanese study indicated that the use of PPI was significantly related to PD-related peritonitis [15].

Our study concluded that PD patients using PPIs were more likely to suffer from the PD-related peritonitis event. The pathophysiological mechanism was still under investigation. In addition to the several mechanisms mentioned above, that is, PPIs promoted the growth and colonization of intestinal bacteria by reducing the pH value of the gastrointestinal tract and directly affected the susceptibility of the body to the inflammatory cells. PPIs can also promote the occurrence of peritonitis by affecting the types of gastrointestinal flora. Genetic sequencing revealed that PPIs significantly increased certain bacterial groups, including Streptococcus and Enterococcus, which were risk factors for intestinal disease [38]. Takagi T *et al.* found that the population of Faecalibacterium genera among PPI users was excessively reduced [21]. Besides, studies have found that butyrate, a product of the Faecalibacterium, enhanced the intestinal mucosal barrier function and immune function [39]. The combination of these factors may explain the use of PPIs to allow bacteria to easily translocate through the damaged barrier of the intestine to the peritoneal cavity to cause infection [11].

Elderly PD patients had multiple comorbidities, increased catabolism, high protein energy consumption, severe daily protein loss through PD, and were more prone to infection events [40]. The most common infectious event in PD patients was peritonitis. The same is true in our study, where the incidence of peritonitis was 26.8% higher than the incidence of pneumonia at 12.4%. In addition, many retrospective reports have identified diabetes mellitus, old age, comorbidities, and hypoalbuminemia as independent risk factors for PD-related peritonitis, which was why peritonitis was more likely to occur [41].

Our article has several advantages. First of all, our study included long-term PD populations from two large hospitals, so our sample size was large and the average follow-up time was long. Moreover, we used the statistical method of IPTW to eliminate the bias caused by confounding factors. In addition, we defined the use of PPI as the use of drugs for at least 1 week to prevent the protopathic bias to avoid reverse causality. Finally, we found that the use of PPIs may increase the incidence of first pneumonia and PD-related peritonitis in PD population. Although the relationship between PPIs and PD-related peritonitis is still controversial, considering the differences in intestinal flora caused by dietary habits, living environment and genes between Asian and Western populations [42, 43]. it may further affect the occurrence of peritonitis [11]. So our study has certain clinical significance [15].

There were three main limitations in our article. First, we did not record the type, dose and specific course of PPIs in our data, nor did we record the relevant information of over-the-counter PPIs drugs, so the impact of PPIs may be underestimated. Second, we did not include the types of microorganisms that cause peritonitis infection, so it was impossible to study the relationship between flora and peritonitis. Third, our study was a retrospective study, prone to bias associated with confounding variable adjustment. Although our study used propensity matching to control for known confounders as much as possible, such biases may still exist if there were unmeasured or unknown confounders.

## Conclusions

The results of this population-based retrospective cohort study indicate that PPIs may be associated with an increased risk of first pneumonia and PD-related peritonitis events in PD patients, and nephrologists should be cautious when prescribing PPIs. Prospective clinical trials are needed to further clarify the association of PPIs with increased risk of infection as a guideline for clinicians.

## Data Availability

The data was obtained with the consent of all centers, and the data in this study are true and reliable.

## References

[1] US Renal Data System. USRDS 2006 annual data report. Bethesda, MD: National Institutes of Health, National Institute of Diabetes and Digestive and Kidney Diseases; 2006.

[2] Ghali JR, Bannister KM, Brown FG, et al. Microbiology and outcomes of peritonitis in Australian peritoneal dialysis patients. Perit Dial Int. 2011;31(6):651–662. 21719685 10.3747/pdi.2010.00131

[3] Pérez Fontan M, Rodríguez-Carmona A, García-Naveiro R, et al. Peritonitis-related mortality in patients undergoing chronic peritoneal dialysis. Perit Dial Int. 2005;25(3):274–284. 15981776

[4] Davenport A. Peritonitis remains the major clinical complication of peritoneal dialysis: the london, UK, peritonitis audit 2002-2003. Perit Dial Int. 2009;29(3):297–302. 19458302

[5] Szeto CC, Wong TY, Chow KM, et al. Are peritoneal dialysis patients with and without residual renal function equivalent for survival study? Insight from a retrospective review of the cause of death. Nephrol Dial Transplant. 2003;18(5):977–982. 12686674 10.1093/ndt/gfg027

[6] Brown MC, Simpson K, Kerssens JJ, et al. Scottish renal registry. Peritoneal dialysis-associated peritonitis rates and outcomes in a national cohort are not improving in the post-millennium (2000-2007. Perit Dial Int. 2011;31(6):639–650. 21804138 10.3747/pdi.2010.00185

[7] Boudville N, Kemp A, Clayton P, et al. Recent peritonitis associates with mortality among patients treated with peritoneal dialysis. J Am Soc Nephrol. 2012;23(8):1398–1405. 22626818 10.1681/ASN.2011121135 PMC3402287

[8] Li PK, Szeto CC, Piraino B, et al. ISPD peritonitis recommendations: 2016 update on prevention and treatment. Perit Dial Int. 2016;36(5):481–508. 27282851 10.3747/pdi.2016.00078 PMC5033625

[9] Lambert AA, Lam JO, Paik JJ, et al. Risk of community-acquired pneumonia with outpatient proton-pump inhibitor therapy: a systematic review and Meta-analysis. PLoS One. 2015;10(6):e0128004. 26042842 10.1371/journal.pone.0128004 PMC4456166

[10] Herzig SJ, Howell MD, Ngo LH, et al. Acid-suppressive medication use and the risk for hospital-acquired pneumonia. JAMA. 2009;301(20):2120–2128. 19470989 10.1001/jama.2009.722

[11] Zhong HJ, Lin D, Lu ZY, et al. Use of gastric-acid suppressants may be a risk factor for enteric peritonitis in patients undergoing peritoneal dialysis: a Meta-analysis. J Clin Pharm Ther. 2019;44(2):209–215. 30332507 10.1111/jcpt.12769

[12] Kawarazaki H, Nakashima A, Furusho M, et al. A questionnaire on prescription patterns of proton pump inhibitors for hemodialysis patients in Japan. Clin Exp Nephrol. 2020;24(6):565–572. 32147803 10.1007/s10157-020-01866-z

[13] Li PK, Szeto CC, Piraino B, et al. Peritoneal dialysis-related infections recommendations: 2010 update [published correction appears in Perit Dial Int. 2011 Sep-Oct;31(5):512]. Perit Dial Int. 2010;30(4):393–423. 20628102 10.3747/pdi.2010.00049

[14] Min YW, Lim KS, Min BH, et al. Proton pump inhibitor use significantly increases the risk of spontaneous bacterial peritonitis in 1965 patients with cirrhosis and ascites: a propensity score matched cohort study. Aliment Pharmacol Ther. 2014;40(6):695–704. 25078671 10.1111/apt.12875

[15] Maeda S, Yamaguchi M, Maeda K, et al. Proton pump inhibitor use increases the risk of peritonitis in peritoneal dialysis patients. PLoS One. 2019;14(11):e0224859. 31697753 10.1371/journal.pone.0224859 PMC6837385

[16] Malfertheiner P, Chan FK, McColl KE. Peptic ulcer disease. Lancet. 2009;374(9699):1449–1461. 19683340 10.1016/S0140-6736(09)60938-7

[17] Lau JY, Barkun A, Fan DM, et al. Challenges in the management of acute peptic ulcer bleeding. Lancet. 2013;381(9882):2033–2043. 23746903 10.1016/S0140-6736(13)60596-6

[18] Ho SW, Hsieh MJ, Yang SF, et al. Risk of stroke-associated pneumonia with Acid-Suppressive drugs: a population-based cohort study. Medicine (Baltimore. 2015;94(29):e1227. ). 26200649 10.1097/MD.0000000000001227 PMC4603020

[19] Lin WL, Muo CS, Lin WC, et al. Association of increased risk of pneumonia and using proton pump inhibitors in patients with type II diabetes mellitus. Dose Response. 2019;17(2):1559325819843383. 31080379 10.1177/1559325819843383 PMC6498779

[20] Takagi T, Naito Y, Inoue R, et al. The influence of long-term use of proton pump inhibitors on the gut microbiota: an age-sex-matched case-control study. J Clin Biochem Nutr. 2018;62(1):100–105. 29371761 10.3164/jcbn.17-78 PMC5773837

[21] Imhann F, Bonder MJ, Vich Vila A, et al. Proton pump inhibitors affect the gut microbiome. Gut. 2016;65(5):740–748. 26657899 10.1136/gutjnl-2015-310376 PMC4853569

[22] Williams C, McColl KE. Review article: proton pump inhibitors and bacterial overgrowth. Aliment Pharmacol Ther. 2006;23(1):3–10. 10.1111/j.1365-2036.2006.02707.x 16393275

[23] Altman KW, Waltonen JD, Tarjan G, et al. Human lung mucous glands manifest evidence of the H+/K+-ATPase proton pump. Ann Otol Rhinol Laryngol. 2007;116(3):229–234. 17419528 10.1177/000348940711600311

[24] Laheij RJ, Sturkenboom MC, Hassing RJ, et al. Risk of community-acquired pneumonia and use of gastric acid-suppressive drugs. JAMA. 2004;292(16):1955–1960. 15507580 10.1001/jama.292.16.1955

[25] Schuijt TJ, Lankelma JM, Scicluna BP, et al. The gut microbiota plays a protective role in the host defence against pneumococcal pneumonia. Gut. 2016;65(4):575–583. 26511795 10.1136/gutjnl-2015-309728 PMC4819612

[26] Jackson MA, Goodrich JK, Maxan ME, et al. Proton pump inhibitors alter the composition of the gut microbiota. Gut. 2016;65(5):749–756. 26719299 10.1136/gutjnl-2015-310861 PMC4853574

[27] de Jager CP, Wever PC, Gemen EF, et al. Proton pump inhibitor therapy predisposes to community-acquired Streptococcus pneumoniae pneumonia. Aliment Pharmacol Ther. 2012;36(10):941–949. 23034135 10.1111/apt.12069

[28] Mikawa K, Akamatsu H, Nishina K, et al. The effects of cimetidine, ranitidine, and famotidine on human neutrophil functions. Anesth Analg. 1999;89(1):218–224. 10389808 10.1097/00000539-199907000-00040

[29] Zedtwitz-Liebenstein K, Wenisch C, Patruta S, et al. Omeprazole treatment diminishes intra- and extracellular neutrophil reactive oxygen production and bactericidal activity. Crit Care Med. 2002;30(5):1118–1122. 12006811 10.1097/00003246-200205000-00026

[30] Aybay C, Imir T, Okur H. The effect of omeprazole on human natural killer cell activity. Gen Pharmacol. 1995;26(6):1413–1418. 7590140 10.1016/0306-3623(94)00301-3

[31] Scaringi L, Cornacchione P, Fettucciari K, et al. Activity inhibition of cytolytic lymphocytes by omeprazole. Scand J Immunol. 1996;44(3):204–214. 8795713 10.1046/j.1365-3083.1996.d01-300.x

[32] Bateman BT, Bykov K, Choudhry NK, et al. Type of stress ulcer prophylaxis and risk of nosocomial pneumonia in cardiac surgical patients: cohort study. BMJ. 2013;347:f5416. 24052582 10.1136/bmj.f5416 PMC3777797

[33] Haas CM, Maywald M, Goetzenich A, et al. Proton-pump inhibitors elevate infection rate in cardiothoracic surgery patients by influencing PMN function in vitro and in vivo. J Leukoc Biol. 2018;103(4):777–788. 29350834 10.1002/JLB.5A0417-143R

[34] Xu HB, Wang HD, Li CH, et al. Proton pump inhibitor use and risk of spontaneous bacterial peritonitis in cirrhotic patients: a systematic review and Meta-analysis. Genet Mol Res. 2015;14(3):7490–7501. 26214428 10.4238/2015.July.3.25

[35] Yu T, Tang Y, Jiang L, et al. Proton pump inhibitor therapy and its association with spontaneous bacterial peritonitis incidence and mortality: a Meta-analysis. Dig Liver Dis. 2016;48(4):353–359. 26795544 10.1016/j.dld.2015.12.009

[36] Kwon JE, Koh SJ, Chun J, et al. Effect of gastric acid suppressants and prokinetics on peritoneal dialysis-related peritonitis. World J Gastroenterol. 2014;20(25):8187–8194. 25057226 10.3748/wjg.v20.i25.8187 PMC4081691

[37] Caravaca F, Ruiz-Calero R, Dominguez C. Risk factors for developing peritonitis caused by micro-organisms of enteral origin in peritoneal dialysis patients. Perit Dial Int. 1998;18(1):41–45. 9527028

[38] Naito Y, Kashiwagi K, Takagi T, et al. Intestinal dysbiosis secondary to Proton-Pump inhibitor use. Digestion. 2018;97(2):195–204. 29316555 10.1159/000481813

[39] Liu H, Wang J, He T, et al. Butyrate: a Double-Edged sword for health? Adv Nutr. 2018;9(1):21–29. 29438462 10.1093/advances/nmx009 PMC6333934

[40] Go AS, Chertow GM, Fan D, et al. Chronic kidney disease and the risks of death, cardiovascular events, and hospitalization. N Engl J Med. 2004;351(13):1296–1305. 15385656 10.1056/NEJMoa041031

[41] Li PK, Chow KM, Van de Luijtgaarden MW, et al. Changes in the worldwide epidemiology of peritoneal dialysis. Nat Rev Nephrol. 2017;13(2):90–103. 28029154 10.1038/nrneph.2016.181

[42] Chen L, Zhang YH, Huang T, et al. Gene expression profiling gut microbiota in different races of humans. Sci Rep. 2016;6:23075. 26975620 10.1038/srep23075 PMC4791684

[43] Brooks AW, Priya S, Blekhman R, et al. Gut microbiota diversity across ethnicities in the United States. PLoS Biol. 2018;16(12):e2006842. 30513082 10.1371/journal.pbio.2006842 PMC6279019

